# Structures of *Saccharomyces cerevisiae* d-arabinose dehydrogenase Ara1 and its complex with NADPH: implications for cofactor-assisted substrate recognition

**DOI:** 10.1107/S1744309113026857

**Published:** 2013-10-26

**Authors:** Xiao-Qian Hu, Peng-Chao Guo, Jin-Di Ma, Wei-Fang Li

**Affiliations:** a College of Life and Environment Science, Huangshan University, Huangshan, Anhui 245041, People’s Republic of China; b Hefei National Laboratory for Physical Sciences at the Microscale and School of Life Sciences, University of Science and Technology of China, Hefei, Anhui 230027, People’s Republic of China

**Keywords:** *Saccharomyces cerevisiae*, Ara1, aldo–keto reductases, substrate-binding pocket, induced fit

## Abstract

The primary role of yeast Ara1, previously mis-annotated as a d-arabinose dehydrogenase, is to catalyze the reduction of a variety of toxic α,β-dicarbonyl compounds using NADPH as a cofactor at physiological pH levels. Here, crystal structures of Ara1 in apo and NADPH-complexed forms are presented at 2.10 and 2.00 Å resolution, respectively. Ara1 exists as a homodimer, each subunit of which adopts an (α/β)_8_-barrel structure and has a highly conserved cofactor-binding pocket. Structural comparison revealed that induced fit upon NADPH binding yielded an intact active-site pocket that recognizes the substrate. Moreover, the crystal structures combined with computational simulation defined an open substrate-binding site to accommodate various substrates that possess a dicarbonyl group.

## Abstract 2

Here, crystal structures of Ara1 in apo and NADPH-complexed forms are presented at 2.10 and 2.00 Å resolution, respectively.

## 1. Introduction

d-Erythroascorbic acid (eAsA) is a major antioxidant that is produced during the metabolic processes in several fungi, such as *Saccharo­myces cerevisiae* (Nick *et al.*, 1986 ▶), *Neurospora crassa* (Dumbrava & Pall, 1987 ▶) and *Candida* (Pall & Robertson, 1988 ▶), and corresponds to ascorbic acid (AsA) in animals (Meister, 1994 ▶) and plants (Asada, 1999 ▶). The biosynthetic pathway of eAsA is composed of two successive reactions that are catalyzed by d-arabinose dehydrogenase (Ara; Kim *et al.*, 1998 ▶) and d-arabinono-γ-lactone oxidase (Alo; Huh *et al.*, 1998 ▶). Two *S. cerevisiae* genes, *YBR149W* and *YMR041C*, have been annotated as encoding two types of Ara: Ara1 and Ara2, respectively (Kim *et al.*, 1998 ▶). However, the *K*_m_ value of Ara1 towards d-arabinose is about 160 m*M*, which is 200 times that of Ara2 (0.78 m*M*). The high Michaelis constant suggests that Ara1 might possess ineffective d-arabinose dehydrogenase activity since the intracellular d-arabinose concentration in yeast is far lower than 100 m*M* (Amako, Fujita, Iwamoto *et al.*, 2006 ▶). Moreover, the results of deletion of the *ARA1* or *ARA2* gene further confirmed that Ara2, and not Ara1, contributes the majority of the production of AsA from d-arabinose (Amako, Fujita, Shimohata *et al.*, 2006 ▶; Amako, Fujita, Iwamoto *et al.*, 2006 ▶).

Ara1 was subsquently re-annotated as an α,β-dicarbonyl reductase which belongs to the aldo–keto reductase (AKR) family (van Bergen *et al.*, 2006 ▶). That is, Ara1 catalyzes the reduction of α,β-dicarbonyl compounds such as methylglyoxal, diacetyl and pentanedione, which are known to be toxic metabolic by-products. These reactive compounds react with proteins and nucleic acids, leading to mutagenesis and damage (Kovacic & Cooksy, 2005 ▶; Wondrak *et al.*, 2002 ▶). In addition, the presence of the metabolite diacetyl in beverages such as beer gives a butterscotch-like aroma and an unpleasant flavour. Thus, it would be useful to engineer a strain of yeast that could enzymatically reduce diacetyl to acetoin (2-hydroxy-3-butanone), a more flavour-neutral compound in beer (van Bergen *et al.*, 2006 ▶). In the presence of saturated NADPH, the *K*_m_ values of Ara1 towards 2,3-pentanedione, diacetyl and methylglyoxal at pH 4–5 are 4.2, 5.0 and 14.3 m*M*, respectively. The *k*_cat_ values towards these substrates are in the range 4.4–5.9 s^−1^, which is fast enough to catalyze degradation of these toxic compounds (van Bergen *et al.*, 2006 ▶). Moreover, proteomics results demonstrated a twofold increase of Ara1 expression in response to H_2_O_2_ stimuli (Godon *et al.*, 1998 ▶). Microarray data showed that environmental stresses, including heat shock and oxidative stress, markedly stimulate up-regulation of *ara1* transcription in yeast cells (Gasch *et al.*, 2000 ▶). It is suggested that the primary role of Ara1 is to reduce a variety of toxic aldehydes and ketones produced during stress.

Although some homologous proteins to Ara1, such as human Akr1b10 (Δ4-3-ketosteroid 5β-reductase; PDB entry 3cav; Faucher *et al.*, 2008 ▶) and murine FR-1 (fibroblast growth factor 1; PDB entry 1frb; Wilson *et al.*, 1995 ▶), which belong to the AKR family have been characterized, the crystal structure of yeast Ara1 is still not available. Here, we determined the first crystal structures of Ara1: in the apo form at 2.10 Å resolution and complexed with the coenzyme NADPH at 2.00 Å resolution. These two structures enabled us to illustrate an induced fit upon NADPH binding and to define an accommodative substrate-binding site which would detoxify a broad spectrum of substrates.

## 2. Materials and methods

### 2.1. Cloning, expression and purification of Ara1 in *Escherichia coli*

The coding sequence of *ARA1*/*YBR149W* was cloned into a pET28a-derived vector. This construct adds a hexahistidine tag to the N-terminus of the recombinant protein, which was overexpressed in *E. coli* BL21 (DE3) strain (Novagen, Madison, Wisconsin, USA) using 2×YT (yeast extract and tryptone) culture medium. The cells were induced with 0.2 m*M* isopropyl β-d-1-thiogalactopyranoside (IPTG) at 289 K for 20 h when the OD_600 nm_ reached 0.6. The cells were harvested by centrifugation at 8000*g* for 10 min and resuspended in lysis buffer (20 m*M* Tris–HCl pH 8.0, 200 m*M* NaCl). After 5 min of sonication and centrifugation at 12 000*g* for 25 min, the supernatant containing the soluble target protein was collected and loaded onto an Ni–NTA column (GE Healthcare) equilibrated with binding buffer (20 m*M* Tris–HCl pH 8.0, 200 m*M* NaCl). The target protein was eluted with 250 m*M* imidazole buffer and loaded onto a Superdex 200 column (GE Healthcare) equilibrated with 20 m*M* Tris–HCl pH 8.0, 50 m*M* NaCl. Fractions containing the target protein were pooled and concentrated to 20 mg ml^−1^. The purity of the protein was estimated by SDS–PAGE and the protein sample was stored at 193 K.

### 2.2. Crystallization, data collection, structure solution and refinement of Ara1 and the Ara1–NADPH complex

Crystals of Ara1 were obtained at 289 K using the hanging-drop vapour-diffusion technique by mixing 1 µl protein solution at 10 mg ml^−1^ with an equal volume of reservoir solution (25% polyethylene glycol 3350, 0.1 *M* HEPES pH 7.5).

Crystals of the Ara1–NADPH complex were obtained by co-crystallization with 5 m*M* NADPH in 25% polyethylene glycol 3350, 0.1 *M* bis-tris pH 6.5, 0.05 *M* CaCl_2_.

The crystals were flash-cooled in liquid nitrogen and data sets were collected at a radiation wavelength of 0.9795 Å on beamline BL17U at Shanghai Synchrotron Radiation Facility (SSRF) at 100 K using an MX-225 CCD detector (MAR Research). Data processing and scaling were performed using the *HKL*-2000 package (Otwinowski & Minor, 1997 ▶). The crystal structure of Ara1 was determined by the molecular-replacement method with *MOLREP* using the coordinates of human AKR in complex with NADPH and inhibitor (PDB entry 1zua; Gallego *et al.*, 2007 ▶) as the search model. Refinement was carried out using the maximum-likelihood method in *REFMAC* (Murshudov *et al.*, 2011 ▶) and the interactive rebuilding process was performed using *Coot* (Emsley & Cowtan, 2004 ▶). The overall model quality was assessed with *MolProbity* (Chen *et al.*, 2010 ▶). Atomic coordinates and structure factors have been deposited in the Protein Data Bank (PDB; http://www.rcsb.org) under accession codes 4ijc and 4ijr. The crystallo­graphic parameters of the structure are listed in Table 1 ▶. All structural figures were prepared using *PyMOL* (http://www.pymol.org).

## 3. Results and discussion

### 3.1. Overall structure

**Figure 1:**
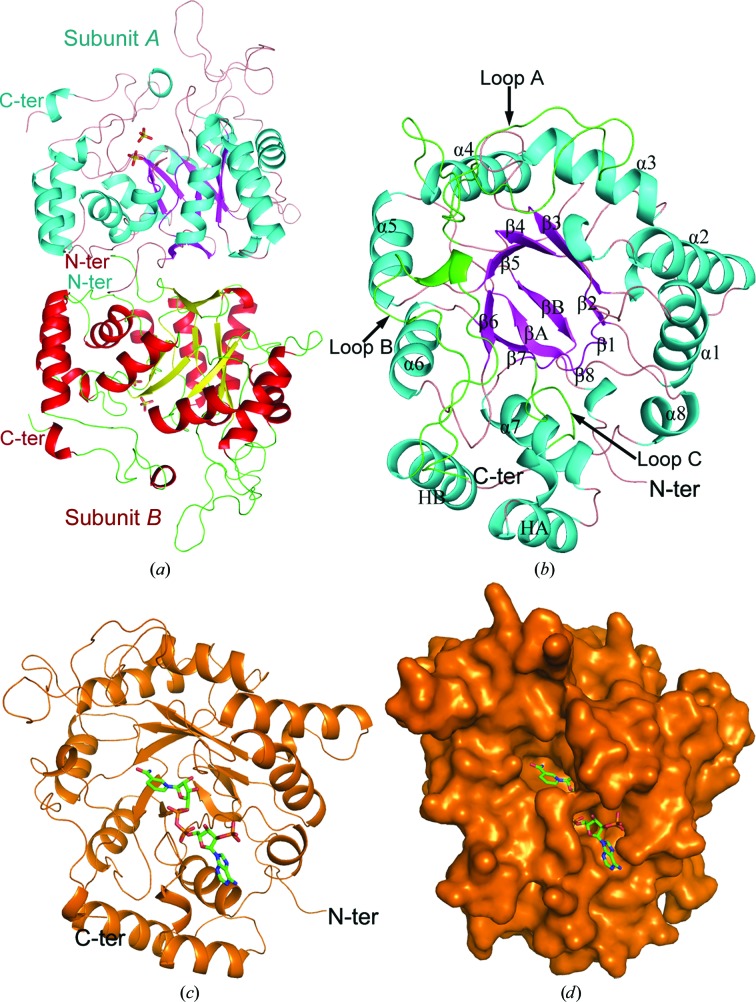
Overall structure. Schematic representation of (*a*) the Ara1 dimer and (*b*) the Ara1 monomer. (*c*) Cartoon representation and (*d*) molecular surface of the Ara1–NADPH complex. NADPH is shown as green sticks. All figures were drawn using *PyMOL*.

The asymmetric unit contains a dimer of Ara1 with an interface area of 1030 Å^2^. The two subunits are very similar, with an overall root-mean-square deviation (r.m.s.d.) of 0.13 Å over 296 C^α^ atoms (Fig. 1 ▶ *a*). Gel-filtration chromatography also indicated the existence of Ara1 as a dimer in solution. The dimeric interface is mainly mediated by strands βA, βB and two loops (Met15–Tyr24 and Lys91–Leu96) in each subunit and contains eight hydrogen bonds and 111 non-bonded contacts, which include hydrophobic interactions and salt bridges.

Each Ara1 subunit adopts an (α/β)_8_-barrel topology or TIM-barrel (Banner *et al.*, 1975 ▶) motif (Fig. 1 ▶ *b*). As in other AKR structures (Wilson *et al.*, 1992 ▶), the TIM barrel is mainly composed of eight parallel β-strands, and each β-strand alternates with an α-helix running antiparallel to the strand. The two antiparallel β-strands (βA and βB) at the N-terminus cover the bottom of the barrel. The top of the barrel is partially covered by three large exposed loops, loops A (between β4 and α4; Glu133–Tyr166), B (between β7 and α7; Tyr241–Pro248) and C (at the carboxyl-terminus; Lys316–Leu342), and two α-­helices, helix A (Pro254–Ile263) between β7 and α7 and helix B (Lys303–Lys315) between helix 8 and loop C (Fig. 1 ▶ *b*).

### 3.2. Induced-fit NADPH binding

**Figure 2:**
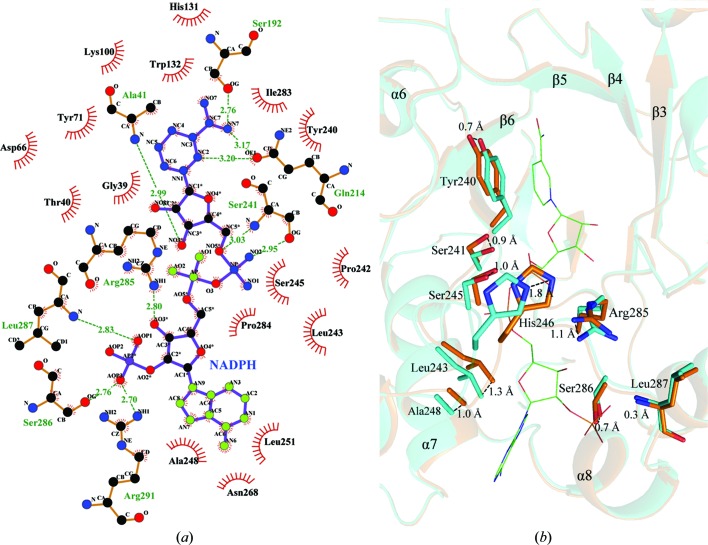
NADPH-binding site. (*a*) Interactions between NADPH and Ara1. (*b*) Induced fit upon NADPH binding. Ara1 is shown in cyan and the Ara1–NADPH complex is shown in orange. NADPH is shown in green lines and the interacting residues are shown as sticks.

The structure of the Ara1–NADPH complex showed that a molecule of NADPH binds at the carboxyl edge of the β-strands of the barrel in an extended conformation (Figs. 1 ▶ *c* and 1 ▶ *d*). In detail, the adenine ring of NADPH is stabilized by the main chains of Ala249 and Ser250 and the side chains of Ala248, Leu251, Asn268 and Arg291 *via* van der Waals interactions. The phosphate group of the adenosine ribose is fixed by Ser286 O^γ^, Leu287 N and Arg291 N^η1^ and the hydroxyl group of the adenosine ribose is stabilized by Arg285 N^η1^ through hydrogen bonds. The pyrophosphate is threaded through a short tunnel, with one side occupied by Ser241–His246. The other side is lined with Ile283, Pro284 and Arg285. The pyrophos­phate group forms two hydrogen bonds to Ser241 N and O^γ^. One hydroxyl group of the nicotinamide ribose makes a hydrogen bond to Ala41 N. The nicotinamide moiety accommodates a wider cavity and forms hydrogen bonds to Gln214 O^∊1^ and Ser192 O^γ^ (Fig. 2 ▶ *a*). Superposition of apo-form Ara1 on the the Ara1–NADPH complex yields an r.m.s.d. of 0.25 Å over 297 C^α^ atoms. The major conformational change results from the induced fit upon NADPH binding. In order to accommodate the cofactor, loop B (Tyr240–Pro249) and a short segment (Ile283–Arg291) near the C-terminal end shift towards each other and lead to a narrower cleft (Fig. 2 ▶ *b*). For example, Leu243, Ser245, His246 and Ala248 shift by 1.8, 1.0, 1.3 and 1.0 Å, respectively, whereas the phenolic ring of Tyr240 and the hydroxyl group of Ser241 shift by about 0.7 and 0.9 Å, respectively, leaving space for the NADPH nicotinamide moiety. On the other side, Arg285, Ser286 and Leu287 also shift by about 1.1, 0.7 and 0.3 Å, respectively, to stabilize the adenosine ribose of NADPH (Fig. 2 ▶ *b*).

### 3.3. The proposed binding sites for α,β-dicarbonyl compounds

**Figure 3:**
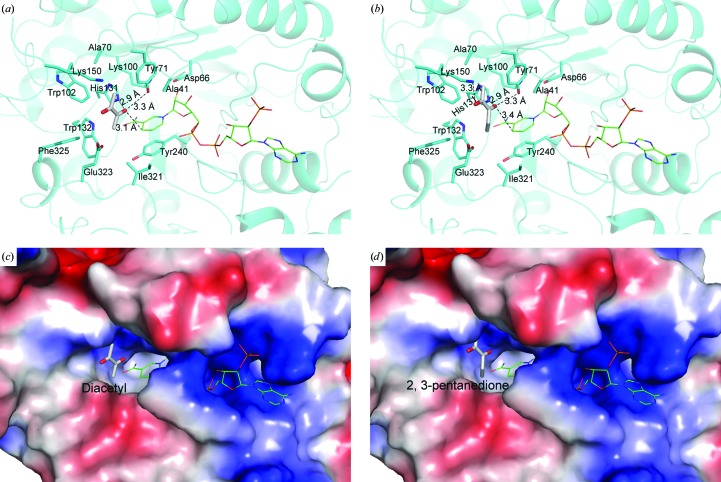
A docking model of Ara1 complexed with diacetyl or 2,3-pentanedione. (*a*, *b*) Binding patterns of (*a*) diacetyl and (*b*) 2,3-pentanedione. (*c*, *d*) Surface potentials of Ara1 complexed with (*c*) diacetyl and (*d*) 2,3-pentanedione. Residues are shown as cyan sticks and diacetyl or 2,3-pentanedione as grey sticks. NADPH is shown as thinner sticks. Hydrogen bonds are shown as black dashes.

**Figure 4:**
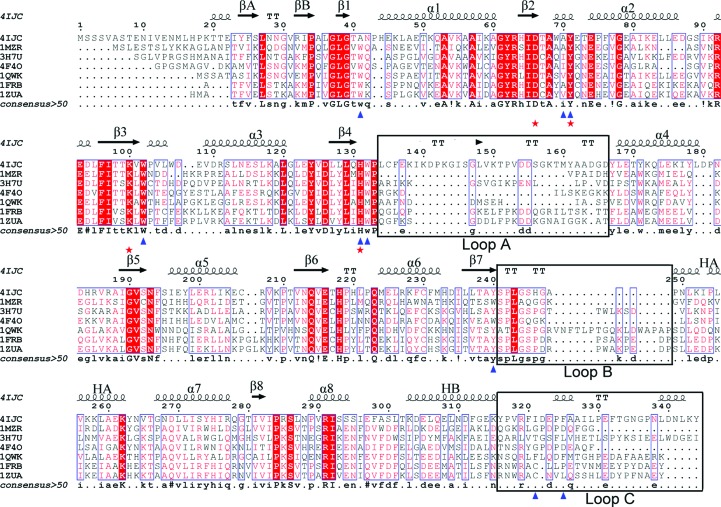
Multiple sequence alignment of proteins in the aldo–keto reductase (AKR) family. Proteins are represented by their PDB codes: 4ijc, *Saccharomyces cerevisiae* Ara1 (NP_009707.3); 1mzr, *Escherichia coli* Dkga (NP_417485.4; Jeudy *et al.*, 2006 ▶); 3h7u, *Arabidopsis thaliana* NADP-linked oxidoreductase (NP_001031505.1; Simpson *et al.*, 2009 ▶); 4f4o, *Leishmania braziliensis* Ara1 (XP_001685202.1; Andersen *et al.*, 2012 ▶); 1qwk, *Caenorhabditis elegans* Ara1 (NP_509242.1; Southeast Collaboratory for Structural Genomics, unpublished work); 1frb, *Mus musculus* aldose reductase (NP_032038.1; Wilson *et al.*, 1995 ▶); 1zua, *Homo sapiens* Akr1b10 (NP_064695.3; Gallego *et al.*, 2007 ▶). The secondary-structure elements of Ara1 (PDB entry 4ijc) are shown at the top. Residues involved in substrate binding are labelled with blue triangles and catalytic residues are marked with red stars. The alignment was performed with *ClustalW* (Larkin *et al.*, 2007 ▶) and *ESPript* (Gouet *et al.*, 1999 ▶).

The substrate-binding pocket of AKRs has been proposed and is reported to be close to the nicotinamide moiety of the NADPH cofactor (Jez *et al.*, 1997 ▶; Di Costanzo *et al.*, 2009 ▶; Wilson *et al.*, 1992 ▶). To clarify the structural basis of the catalysis driven by Ara1, we attempted to obtain a crystal of the tertiary complex of Ara1 with NADP^+^ and an α,β-dicarbonyl compound by either co-crystallization or crystal soaking, but were not successful. Therefore, we docked two typical α,β-dicarbonyl substrates, diacetyl and 2,3-pentanedione, into the structure of Ara1–NADPH using *HADDOCK* (de Vries *et al.*, 2010 ▶). The docking program was driven by interaction restraints between the active-site residues of Ara1 and the α,β-dicarbonyl substrate, as defined by *WHISCY* (Adams *et al.*, 2002 ▶) and previously reported by Jez *et al.* (1997 ▶). Docking produced 25 clusters for diacetyl and 32 for 2,3-pentanedione. The results for each substrate were selected as the cluster of lowest energy that satisfied the best interaction restraints. In this mode, the topology of the substrate-binding site resembles an open and accommodative cleft and includes three components: the oxyanion-binding site (Tyr71, His131 and C4 of the nicotinamide ring), residues at the edge of the active site (Ala41, Ala70, Trp102 and Trp132) forming a hydrophobic environment and amino acids from three loops forming the sides of the cleft: loop A contributes to one side (Lys150 and Thr151), with the opposite side being formed by loop B (Tyr240 and His246) and loop C (Ile321, Glu323 and Phe325) (Figs. 3 ▶ *a* and 3 ▶ *b*). The substrate-binding pocket is predominantly hydrophobic, in accord with the generally hydrophobic nature of the dicarbonyl substrates. In the binding mode, all of the substrate packed perpendicular to the nicotinamide ring, with one carbonyl O atom of the α,β-dicarbonyl compound interacting with the side chain of Tyr71, His131 and the nicotinamide ring through hydrogen bonds (about 3.1 Å for diacetyl and 3.4 Å for 2,3-pentanedione; Figs. 3 ▶ *a* and 3 ▶ *b*). Tyr71 was proposed as the catalytic residue (proton donor) based on the proximity required between the C4 position of the nicotinamide ring and the anticipated position of the substrate carbonyl (Wilson *et al.*, 1992 ▶; Jez *et al.*, 1997 ▶). The other carbonyl is exposed towards the outside of the substrate-binding pocket (Figs. 3 ▶ *c* and 3 ▶ *d*). Meanwhile, the conserved hydrophobic residues Ala41, Ala70, Trp102, Trp132, Tyr240, Ile321 and Phe325 form a hydrophobic environment to accommodate the carbon skeleton of the α,β-dicarbonyl compound (Figs. 3 ▶ *a* and 3 ▶ *b*). Sequence analysis reveals that the active-site residues Ala41, Asp66, Ala70, Tyr71, Lys100, Trp102, His131, Trp132, Tyr240, Ile321 and Phe325 are all conserved in the AKR family (Fig. 4 ▶) and possess a common substrate-binding pattern. Analysis of the Ara1 structure shows that the three loop regions (A, B and C) exhibit relatively high *B* factors, and this structural flexibility and plasticity was supposed to be necessary for the recognition of more than one substrate (Jez *et al.*, 1997 ▶). Furthermore, multiple sequence alignment also shows that the composition and length of the amino acids in the three loops (A, B and C) varies (Fig. 4 ▶), which probably determines the substrate specificities of the different AKRs. In conclusion, the open and accommodative substrate-binding site forms a favourable environment for various α,β-dicarbonyl substrates.

### 3.4. A putative catalytic mechanism

The docking results enable us to propose a plausible catalytic mechanism for Ara1. In the apo form, the cofactor-binding pocket and the active site are relatively open and relaxed. Upon binding of NADPH, loop B (Tyr240–Pro249) and a short segment (Ile283–Arg291) near the C-terminal end move towards each other to narrow the cofactor-binding cleft. Meanwhile, the active-site residues form a cavity favourable for substrate binding (Jez *et al.*, 1997 ▶; Wilson *et al.*, 1992 ▶). The substrate in the pocket is correctly positioned by the side chains of Ala70, Tyr71, His131, Trp102 and Trp132, as well as the NADPH nicotinamide ring. An electron immediately transfers from C4 of the nicotinamide to the carbonyl group of the substrate. Upon reduction of the carbonyl group of the α,β-dicarbonyl substrate, the hydrogen bond between the catalytic residue Tyr71 and the carbonyl group of the substrate disappears. With the change in redox state, NADP^+^ may undergo a conformational change accompanied by the opening of the cofactor-binding cleft for release of the product.

## Supplementary Material

PDB reference: Ara1, 4ijc

PDB reference: complex with NADPH, 4ijr

## Figures and Tables

**Table 1:** Data-collection and refinement statistics Values in parentheses are for the highest resolution shell.

	Apo form	NADPH-bound form
Data collection		
Space group	*P*2_1_2_1_2_1_	*P*2_1_
Unit-cell parameters (Å, °)	*a* = 71.90, *b* = 91.57, *c* = 107.64, α = 90.00, β = 90.00, γ = 90.00	*a* = 54.37, *b* = 90.57, *c* = 69.82, α = 90.00, β = 90.08, γ = 90.00
Molecules per asymmetric unit	2	2
Resolution range (Å)	50.00–2.10 (2.18–2.10)	50.00–2.00 (2.07–2.00)
Unique reflections	41412 (4074)	45854 (4460)
Completeness (%)	99.9 (100.0)	98.3 (95.8)
〈*I*/σ(*I*)〉	19.56 (10.00)	21.98 (7.38)
*R*_merge_ † (%)	8.6 (25.7)	4.8 (17.8)
Average multiplicity	7.3	3.8
Structure refinement		
Resolution range (Å)	50.00–2.10 (2.16–2.10)	34.91–2.00 (2.05–2.00)
*R* factor‡/*R*_free_ § (%)	22.3/26.4 (22.7/30.7)	20.6/24.7 (23.8/26.4)
No. of protein atoms	5288	5244
No. of water atoms	338	315
R.m.s.d.¶, bond lengths (Å)	0.007	0.011
R.m.s.d., bond angles (°)	0.985	1.337
Mean *B* factor (Å^2^)	33.9	38.9
Wilson *B* factor (Å^2^)	30.7	32.2
Ramachandran plot†† (%)		
Most favoured	98.0	97.9
Additionally allowed	2.0	1.8
PDB entry	4ijc	4ijr

† *R*_merge_ = 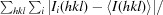 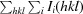, where *I*_*i*_(*hkl*) is the intensity of an observation and 〈*I*(*hkl*)〉 is the mean value for its unique reflection; summations are over all reflections.

‡ *R* factor = 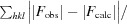 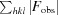, where *F*_obs_ and *F*_calc_ are the observed and calculated structure-factor amplitudes, respectively.

§ *R*_free_ was calculated using 5% of the data, which were excluded from the refinement.

¶ Root-mean-square deviation from ideal values (Engh & Huber, 1991 ▶).

†† Categories as defined by *MolProbity* (Chen *et al.*, 2010 ▶).
